# Fabrication of Lead–Zinc Tailings Sintered Brick and Its Effect Factors Based on an Orthogonal Experiment

**DOI:** 10.3390/ma17102352

**Published:** 2024-05-15

**Authors:** Hang Lin, Rui Li, Su Li

**Affiliations:** School of Resources and Safety Engineering, Central South University, Changsha 410083, China; hanglin@csu.edu.cn (H.L.); 215511001@csu.edu.cn (R.L.)

**Keywords:** lead–zinc tailings, sintered ordinary brick, orthogonal experiment, compressive strength

## Abstract

The existence of lead-zinc tailings threatens the social and ecological environment. The recycling of lead–zinc tailings is important for the all-round green transformation of economic society. In this study, the possibility of fabricating sintered ordinary bricks with lead–zinc tailings was studied based on orthogonal experimentation, and the phase composition and micromorphology of sintered products were analyzed by X-ray diffraction (XRD) and scanning electron microscope (SEM). With lead–zinc tailings as the main material, and clay and fly ash as additives, the effect of clay content, forming pressure, sintering temperature, and holding time on physical properties of sintered bricks was analyzed. The results show that clay content and sintering temperature have a major effect on compressive strength, while sintering temperature and holding time play an important role in water absorption. During sintering, mica, chlorite, and other components in lead–zinc tailings are decomposed to form albite, hematite, maghemite, and anhydrite, which play a role in the strength of bricks. The optimal process parameters were found to be a ratio of lead–zinc tailings:clay:fly ash = 6:3:1, forming pressure of 20 MPa, firing temperature of 1080 °C, and holding time of 60 min. The corresponding compressive strength and water absorption were 34.94 MPa and 16.02%, which meets the Chinese sintered ordinary bricks standard (GB/T 5101-2017).

## 1. Introduction

China’s lead–zinc ore production accounts for more than 40% of the world’s total production. Lead–zinc tailings are the residual parts of lead–zinc ore after grinding and flotation, which are very representative in China’s solid ore waste dumps [1]. Tailings storage is the most direct disposal method at present. The annual production of tailings in China is about 1.5 billion tons, and there are more than 7000 tailings ponds in China. However, after being disturbed, tailings reservoirs may cause geological disasters such as debris flows, landslides, and ground collapse, resulting in heavy casualties and property losses [2,3]. Furthermore, long-term accumulation will lead to the leaching of harmful components containing heavy metal ions such as Pb^2+^, Zn^2+^, and Cd^2+^, threatening the environmental safety of water and soil near the mining area [4]. Therefore, there is an urgent need to carry out research on the reutilization of lead–zinc tailings, which is of great significance to improve resource utilization efficiency, improve environmental quality, and promote the comprehensive green transformation of economic and social development.

At present, the comprehensive utilization of lead–zinc tailings mainly has two aspects. First, lead–zinc tailings are selected as secondary resources to recover the valuable components and improve the recovery rate. The second is the direct utilization of lead–zinc tailings, mainly including the production of building materials and backfill materials. The grades of lead, zinc, sulfur, and fluorite in lead–zinc tailings are relatively high, and these components have a high recovery value. At present, the secondary recovery of lead–zinc tailings is achieved by a variety of processes, including chemical leaching, microbial leaching, magnetizing roasting–magnetic separation, and flotation technology [5,6,7,8,9]. However, the existence of difficult-to-recover metals in tailings will result in secondary tailings, which will also threaten human society. Therefore, the direct utilization of tailings without reselection value should be considered.

Using tailings as the main raw material to produce building materials can not only consume a large quantity of tailings, but also bring good economic benefits to mining enterprises. This direction has already attracted the extensive attention of scholars [10,11]. Guo et al. [12] studied the preparation of ceramic bricks from tungsten tailings. Kim et al. [13] and Wei et al. [14] studied the possibility of fabricating bricks from gold tailings. Luo et al. [15] used iron tailings, sludge, and other materials to prepare sintered bricks, systematically analyzed the influence of many factors on the properties of the sintered bricks, and proposed the optimal process parameters. In addition, other scholars have conducted extensive research on the use of tailings to prepare ceramics [16,17], filling materials [18,19], cement material [20,21,22,23], etc.

Due to the low grade of lead–zinc ore in China, the output of lead–zinc tailings is usually more than ten times that of lead–zinc concentrate. The disposal of lead–zinc tailings has become a key problem restricting the development of the lead–zinc industry. Si, Al, and other elements contained in lead–zinc tailings are essential components of building materials production [24,25]. Therefore, if lead–zinc tailings can be used as a substitute for building materials, this can solve the problem of tailings storage and maximize the effective utilization of resources [26,27]. In recent years, many scholars have carried out research on the use of lead–zinc tailings as raw materials to fabricate building materials. Liu et al. [28,29,30] fabricated foam ceramics from lead–zinc tailings, red mud, and fly ash, and studied the influence of various process parameters on ceramic performance. By studying the geopolymers with lead–zinc tailings, Zhao et al. [31] found that the curing rates of Zn^2+^, Pb^2+^, and Cd^2+^ were all higher than 97.80%, and the leaching concentrations only fluctuated within the limited environmentally acceptable range. In addition, Wang et al. [32] analyzed the fixation behavior of heavy metal ions in the sintering process of lead–zinc tailings brick and found that a high temperature (over 1050 °C) can play a positive role in the fixation of heavy metal ions. Li et al. [33] and Zhang et al. [34] studied the leaching behavior of heavy metal ions when lead–zinc tailings were used as raw materials to prepare building materials. Wang et al. [35] studied the use of lead–zinc tailings to prepare ultra-high-performance concrete and found that the addition of lead–zinc tailings can significantly reduce the early auto-shrinkage of concrete and is conducive to the development of its microstructure. By studying the effect of temperature on the performance of foam ceramics mixed with lead–zinc tailings, Liu et al. [28] found that foam ceramics with sintering temperature at 970 °C had the best performance, with higher porosity (76.2%), higher mechanical strength (5.3 MPa), and lower thermal conductivity (0.21 W/(m K)). In summary, the current use of lead–zinc tailings as raw materials to prepare ceramics, cementing materials, and fillers has been widely reported. However, there are few reports on the fabrication of sintered bricks with lead–zinc tailings.

In this study, lead–zinc tailings were used as primary raw materials, and clay and fly ash as auxiliary materials, to fabricate sintered bricks that reached the highest strength grade in the Chinese sintered ordinary bricks standard (GB/T 5101-2017 [36]). The approximate range of the raw material ratio was determined by the single-factor test. The effects of clay content, forming pressure, sintering temperature, and holding time on the properties of sintered bricks were analyzed based on orthogonal experiments. The brick sintering process was studied with XRD, SEM, and thermogravimetry/differential scanning calorimetry technology (TG-DSC). The research results are expected to realize the secondary utilization of lead–zinc tailings and reduce the environmental and safety threats caused by the accumulation of lead–zinc tailings.

## 2. Materials and Experiments

**Figure 1:**
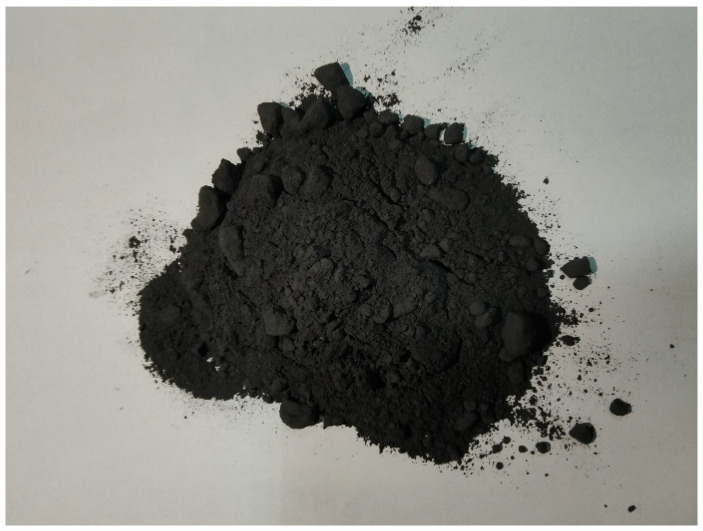
Lead–zinc tailings powder used in this study.

The lead–zinc tailings used in this study are tailings waste residues after reconcentration of tailings, which are from the Sanguikou lead–zinc tailings of Ulat Houqi Zijin Mining Co, Inner Mongolia, China (Figure 1). Clay and fly ash, the auxiliary materials, were purchased from the market.

### 2.1. Physicochemical Properties Test

The phase composition of lead–zinc tailings was determined by XRD (Bruker D8 ADVANCE, Bruker, Mannheim, Germany, angular accuracy is 0.0001°), and the chemical compositions of three raw materials (lead–zinc tailings, clay, and fly ash) were examined by an X-ray fluorescence spectroscopy analyzer (XRF, Bruker S2 PUMA, Bruker, Germany). The thermodynamic characteristics of lead–zinc tailings were analyzed through TG-DSC (Netzsch/STA 449 F5 Jupiter, Netzsch, Germany, balance resolution is 0.1 μg and temperature resolution is 0.001 K). The surface morphology and pore structure of lead–zinc tailings particles at different sintering temperatures were scanned by SEM (MIRA3 LMH eds: One Max 20, Tescan, Czech Republic). A laser particle size analyzer (LPSA, Mastersizer 2000 with Hydro2000M, Malvern, UK) was adopted to analyze the particle size distribution of lead–zinc tailings. According to the provisions of the liquid–plastic limit combined test method in the Standard for geotechnical testing method (GB/T50123-2019 [37]), the plasticity index (the difference between the liquid limited water content and the plastic limited water content) of lead–zinc tailings was measured by a liquid–plastic limit combined device. The particle size should be less than 0.5 mm, and the plasticity index was calculated from the average of three parallel tests. The tailings bodies fabricated with different levels of forming moisture were fully dried and a uniaxial compression test was carried out. The drying performance of lead–zinc tailings with different levels of forming moisture was studied by analyzing the shape and strength changes of the tailings bodies, and then the appropriate forming moisture was determined (the corresponding forming moisture of tailing bodies with a smaller shape change and higher strength).

The uniaxial loading experiments of sintered bricks were carried out on a HUALONG WHY-300/10 test system (Hualong, Shanghai, China, relative error of force is less than 0.5%). The maximum load is 300 kN, and the measurement range is 2–100% fullscale, which meets the experimental requirements. According to the Test Method for Wall Bricks (GB/T2542-2012 [38]), the loading method adopts force control and the loading rate is set at 0.8 kN/s. Based on the Chinese sintered ordinary bricks standard (GB/T 5101-2017 [36]), the strength requirements of sintered bricks are divided into five grades named M10, M15, M20, M25, and M30, and the corresponding uniaxial compressive strengths are 10, 15, 20, 25, and 30 MPa, respectively. The phase composition and microstructure of the sintered bricks with lead–zinc tailings prepared by optimal process parameters were analyzed by XRD and SEM.

### 2.2. Specimen Preparation

**Figure 2:**
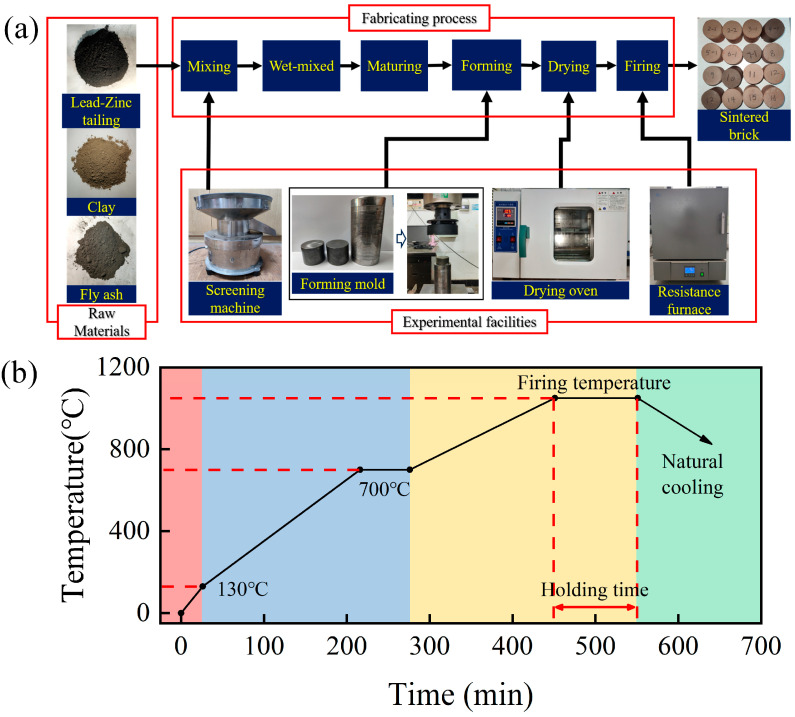
Fabrication of sintered ordinary brick from lead–zinc tailings: (**a**) fabrication process, (**b**) sintering scheme.

Figure 2 shows the process flow and sintering scheme of sintered bricks from lead–zinc tailings. Before preparation began, all raw materials were dried to remove moisture and impurities. As can be seen in Figure 2a, the dried tailings, clay, and fly ash were first screened through a 65-mesh sieve, and then evenly mixed based on the set ratio. According to the set forming moisture, a certain amount of water was added to the mixture to be stirred, and the evenly mixed mixture was put into a sealed bag and aged at room temperature (25 °C) for 24 h. The aged mixture was placed into a Φ50 × 50 mm mold, and compacted to different molding pressures at the loading rate of 0.1 kN/s and maintained for 100 s after forming. The molded bricks were placed at room temperature (25 °C) for 24 h, then placed in a thermostatic drying oven (Supor 101 s, Supor, China, maximum temperature is 300° C and temperature resolution is 0.1 °C) and dried at 105 °C for 12 h. After drying, the bricks were put into a box-type resistance furnace (Yiheng SX2-10-12NP, Yiheng, Shanghai, China, maximum temperature is 1200 °C and temperature resolution is 1 °C) for sintering. After rising to the specified sintering temperature in a certain sintering process, it was kept warm for a certain time, and finally cooled naturally to room temperature. Figure 2b shows the sintering scheme of sintered bricks from lead–zinc tailings, which is divided into four stages: low temperature dehydration stage, stable heating stage, high temperature sintering stage, and natural cooling stage.

### 2.3. Experimental Scheme

The loading experiment was divided into two parts: a single-factor experiment and an orthogonal experiment. For the single-factor experiment, the content of different raw materials is shown in Table 1. The proportion of fly ash was fixed at 10% and the clay content was 0%, 10%, 20%, 30%, and 40%, in order. The forming parameters were as follows: forming moisture was 12.5% and forming pressure was 15 MPa. The sintering parameters were: sintering temperature was 1050 °C and holding time was 60 min. In addition, the results were compared with those of specimens with pure lead–zinc tailings. The ID was given using a-b-c, where a, b, and c, respectively, represented the proportion of tailings, clay, and fly ash.

The orthogonal experiment selected four important parameters in the fabrication process, which were clay content, forming pressure, sintering temperature, and holding time. For each process parameter, four different levels were set, which are shown in Table 2. According to Wang et al. [32], when the sintering temperature is higher than 1050 °C, the leaching rate of Pb and Zn tends to 0. Therefore, the sintering temperature in this experiment was equal to or greater than 1050 °C. According to different process parameters and corresponding levels, the L16 (4^4^) orthogonal test table was used to fabricate sintered brick specimens from lead–zinc tailings. The name of each specimen represents its corresponding process parameters. For example, the corresponding process parameters of specimen A_3_B_1_C_3_D_4_ are that the clay content is 30%, the forming pressure is 15 MPa, the sintering temperature is 1110 °C, and the holding time is 120 min.

## 3. Result Analysis

### 3.1. Raw Materials Characteristics

From Table 3, the lead–zinc tailings used in this study belong to the SiO_2_–Al_2_O_3_–metal oxide system, which is similar to the clay used in traditional sintered bricks. Clay and fly ash were added as auxiliary materials to make sintered bricks with good performance. The chemical compositions of clay and fly ash selected in this study are also shown in Table 3. The clay is brown-yellow and the fly ash is gray. Both of them are mainly powdery particles with a size less than 2 mm, which meet the requirements for fabricating sintered bricks and can be directly mixed with lead–zinc tailings.

**Figure 3:**
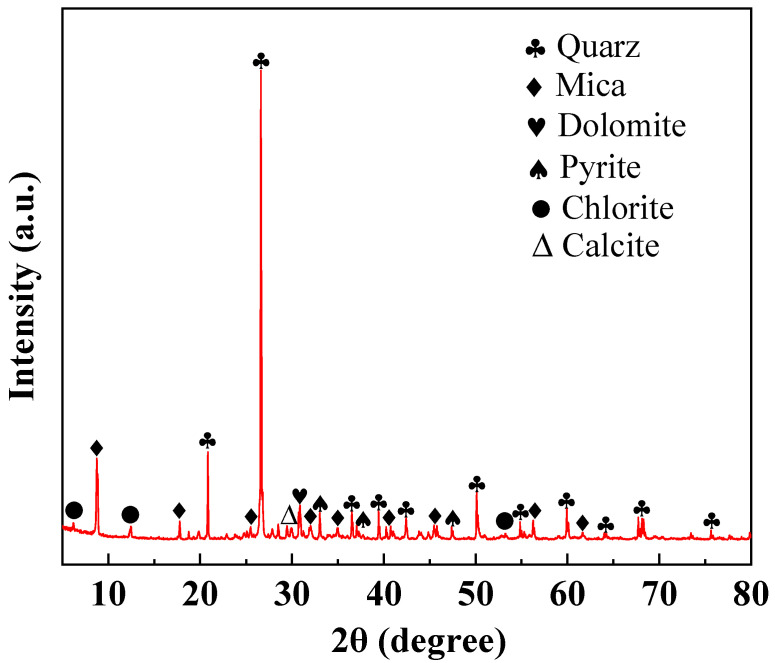
XRD pattern of lead–zinc tailings.

In Figure 3, the XRD pattern of lead–zinc tailings shows that the main phase compositions are quartz, mica, chlorite, and calcite, in addition to dolomite and pyrite, which are consistent with the main chemical composition obtained by XRF analysis. Among them, the diffraction peaks of quartz and mica are sharp and clear, indicating that they have higher content and better crystallinity. Quartz, mica, chlorite, and calcite can be used as raw materials for sintered building materials, which proves the feasibility of applying lead–zinc tailings to the fabrication of sintered ordinary bricks.

**Figure 4:**
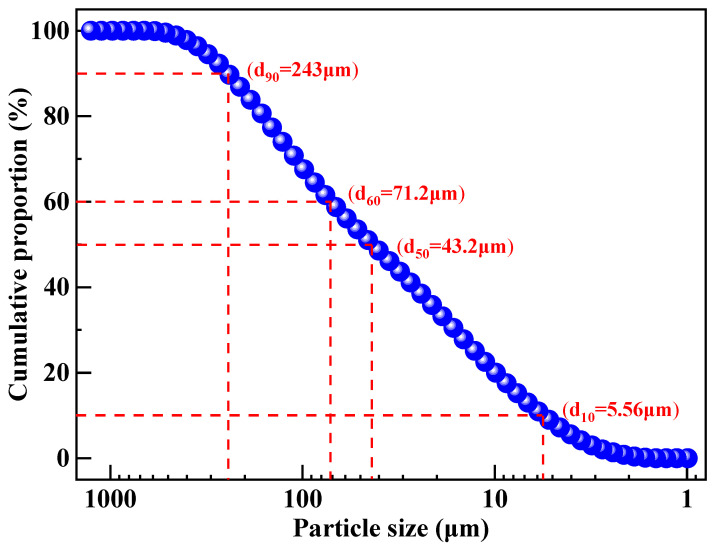
Particle size distribution of lead–zinc tailings.

The particle size distribution of lead–zinc tailings is shown in Figure 4. It can be seen that the particle size of lead–zinc tailings is relatively fine, and the maximum particle size is less than 1 mm. The cumulative proportion of particles with size less than 75 μm reaches 61%, the average particle size is 89.5 μm, and the median particle size is 43.2 μm. The particle size composition of lead–zinc tailings used in this study meets the granularity requirement for preparing sintered ordinary bricks.

**Figure 5:**
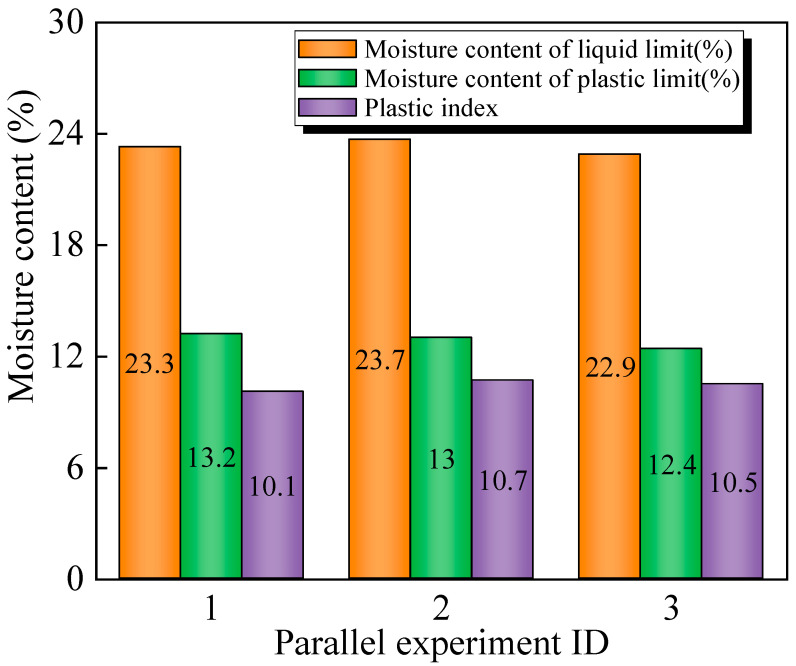
Plasticity index of lead–zinc tailings.

The plasticity index of lead–zinc tailings is shown in Figure 5. It can be seen that the average plasticity index is 10.4, and the index ranges from 7 to 15, which indicates medium plasticity. The plasticity index of tailings meets the requirements of fabricating sintered ordinary brick, and a better molding effect can be achieved when assisted by clay.

**Figure 6:**
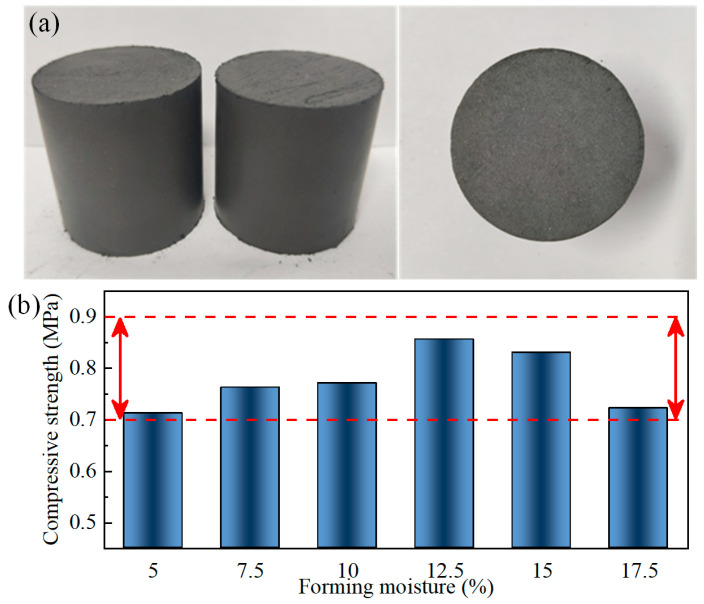
Drying performance of lead–zinc tailings: (**a**) shape comparison; (**b**) relationship between uniaxial compressive strength and forming moisture.

Figure 6 shows the morphology of the tailings body before and after drying, and the strength variation of the tailings body with different levels of forming moisture. It can be seen that the appearance of the tailings body has no obvious change, and the volume shrinkage is basically less than 1%. Forming moisture has a certain influence on the strength of the lead–zinc tailings body. As shown in Figure 6b, with forming moisture ranging from 5% to 17.5%, the uniaxial compressive strength presents a trend of first increasing and then decreasing, and is at a relatively high level in the range of 12.5% to 15%. Generally, the strength variation in the tailings body is relatively stable. When the forming moisture ranges from 12.5% to 15%, the drying performance is relatively good.

### 3.2. Analysis of Single-Factor Experiment

**Figure 7:**
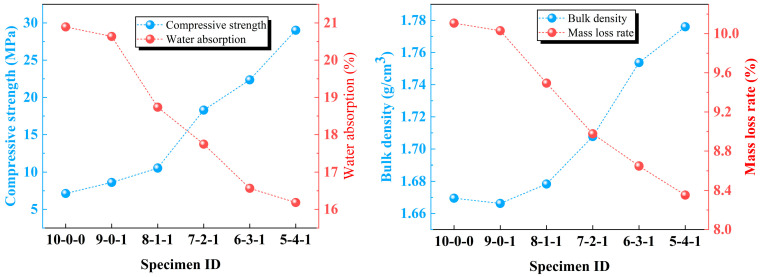
Influence of clay content on performance indices of sintered ordinary bricks from lead–zinc tailings: (**a**) compressive strength and water absorption; (**b**) bulk density and mass loss rate.

Figure 7 shows the influence of clay content on the main performance indices (compressive strength, water absorption, bulk density, and mass loss rate) of sintered ordinary bricks from lead–zinc tailings. In Figure 7a, the addition of fly ash can improve the performance of sintered ordinary bricks, but the improvement is weak. The addition of clay obviously improves the performance of sintered ordinary brick. When the clay content reaches 10%, the compressive strength of the sintered brick is 10.5 MPa and the water absorption rate is 18.7%, which meets the requirements of the MU10 strength level and water absorption in the Chinese sintered ordinary bricks standard (GB/T 5101-2017). With a further increase in clay content, the strength of sintered brick gradually increases, and the water absorption gradually decreases. When the clay content is 30%, the compressive strength of the sintered brick is 22.4 MPa, which reaches the strength level of MU20 in the Chinese sintered ordinary bricks standard (GB/T 5101-2017). Figure 7b shows the influence of raw material ratio on bulk density and mass loss rate of sintered ordinary brick. It can be seen that the addition of fly ash slightly reduces the bulk density, but the subsequent bulk density continues to increase with the increase in clay content. In addition, the mass loss rate of sintered brick decreases continuously, and is less than 15% under six raw material ratios.

### 3.3. Analysis of Orthogonal Experiment

The performance indices of 16 specimens fabricated according to the orthogonal experiment table are shown in Table 4. It can be seen that the compressive strength of specimen A_4_B_2_C_3_D_1_ is the highest, which is 34.43 MPa. When used as a load-bearing brick, it meets the requirements of the MU30 level in the Chinese sintered ordinary bricks standard (GB/T5101-2017). When used as road bricks, it also meets the strength requirements of the MX category in the Chinese fired paving unit standard (GB/T26001-2010 [39]). Considering the requirements of environmental protection and construction in mining areas, the sintered bricks with lead–zinc tailings can replace the pavement bricks around the mining area. For all specimens, except for the slightly larger water absorption of A_1_B_1_C_1_D_1_, A_1_B_2_C_2_D_2_, and A_2_B_1_C_2_D_3_, the water absorption values of other specimens are all less than 17%, and thus meet the requirement. It can be found that the change in process parameters has little influence on mass loss rate and bulk density. Mass loss rate is mainly concentrated in the range of 9%~11%, and the bulk density is between 1.67 g/cm^3^ and 1.81 g/cm^3^.

#### 3.3.1. Mean Value Analysis

**Figure 8:**
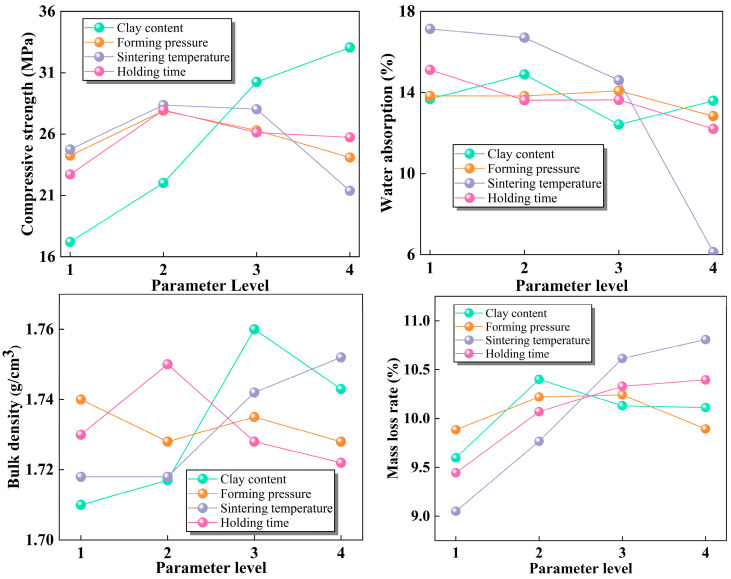
Influence of four process parameters on the average value of performance indices: (**a**) compressive strength; (**b**) water absorption; (**c**) bulk density; (**d**) mass loss rate.

Figure 8 shows the trend of the mean value of performance indices with the variation in each process parameter. In Figure 8a, the strength of sintered ordinary brick is significantly affected by clay content, and the average compressive strength increases linearly with clay content. When clay content is 35%, the uniaxial compressive strength reaches 33 MPa. The average compressive strength of sintered ordinary bricks increases and then decreases with the variation in the three other parameters. The influence of these three process parameters on compressive strength is relatively low, and the maximum points are 1080 °C, 20 MPa, and 60 min, respectively. When the sintering temperature is greater than 1080 °C, the main reason that the strength will gradually decrease is that the increasing sintering temperature leads to the increase in liquid phase content, which results in the degradation of the bricks’ skeleton structure.

In Figure 8b, the mean water absorption of sintered bricks decreased significantly with the variation in sintering temperature, and the decreasing trend is more obvious with the increasing sintering temperature. When the sintering temperature increases from 1050 °C to 1140 °C, the mean water absorption decreases from 17.1% to 6.1%. With the increase in temperature, lead–zinc tailings continue to melt, and liquid phase in sintered brick accumulates, which continuously fills the pores and reduces the porosity of sintered bricks, and finally leads to the weakening of water absorption. The influence of clay content, forming pressure, and holding time on water absorption of sintered bricks is relatively weak, and average water absorption decreases slightly with the increase in the three factor levels.

The variation trends of mean bulk density with the four factor levels are shown in Figure 8c. It is not difficult to see that the bulk density is more affected by sintering temperature and clay content. With the increase in sintering temperature and clay content, the bulk density shows an increasing trend. With the increase in sintering temperature, the amount of liquid phase increases, which filles the pores in sintered bricks and results in the increase in bulk density. When the sintering temperature reaches 1040 °C, the bulk density of sintered brick reaches 1.75 g/cm^3^. Compared with lead–zinc tailings, clay contains a higher proportion of silicon oxides, which melt during the sintering process. The increase in clay content will produce more molten substances, resulting in smaller pores and higher bulk density of sintered bricks. Bulk density was less affected by forming pressure and holding time, which shows a slowly decreasing trend with the increase in these two process parameters.

Figure 8d shows the trend of mass loss rate with the variation in four different factors. During the high-temperature sintering process, raw materials undergo complex physical and chemical reactions. The decomposition of calcite and dolomite produces gas, and water evaporation occurs during the decomposition of mica. These behaviors will result in mass loss of sintered bricks. With the increase in sintering temperature and holding time, the decomposition action will be more complete, and the mass loss rate of sintered brick will increase. When the sintering temperature is 1140 °C, the mass loss rate of sintered brick with lead–zinc tailings reaches 10.8%. With the increase in sintering temperature and holding time, the reaction between chemical components of raw materials will gradually reach completion. This explains why the trend of mass loss rate gradually flattens with the increase in sintering temperature and holding time. In addition, the forming pressure has almost no effect on mass loss rate, which was always maintained near 10% with the increase in forming pressure.

#### 3.3.2. Range Analysis

**Figure 9:**
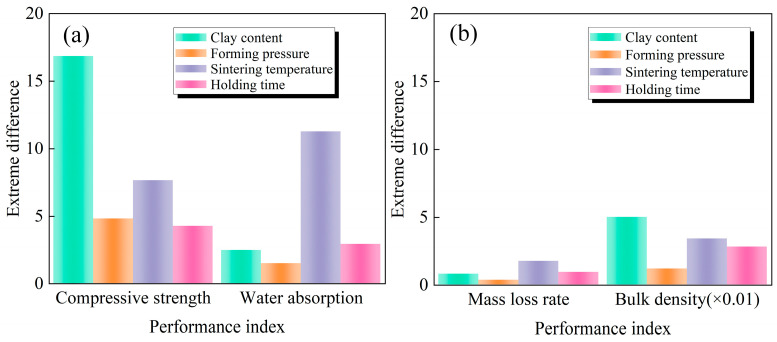
Range of process influences on performance indexes: (**a**) compressive strength and water absorption; (**b**) mass loss rate and bulk density.

Ranges of the influences of the four process parameters on the performance index of sintered bricks are shown in Figure 9. It can be seen that the order of the influence of the four parameters on compressive strength of sintered brick is clay content > sintering temperature > holding time > forming pressure. The order of the influence of the four parameters on water absorption is sintering temperature > holding time > clay content > forming pressure. Careful selection of clay content and sintering temperature can optimize sintered bricks with better performance. The order of the influence of the four parameters on mass loss rate is sintering temperature > holding time > clay content > forming pressure. The sensitivity order of the four parameters on bulk density is clay content > sintering temperature > holding time > forming pressure. However, there is little difference between the range of these four parameters on mass loss rate and bulk density, indicating that the difference in the influence of these four parameters on mass loss rate and bulk density is not obvious. Overall, the sensitivity of the four performance indices to the variation in process parameters is in the order of compressive strength > water absorption > mass loss > volume density

#### 3.3.3. Variance Analysis

The variance analysis of the four process parameters on performance indices of sintered bricks with lead–zinc tailings are shown in Table 5 and Table 6. The significance level α = 0.05 is used in this study, that is, the confidence probability is 95%. When the F-ratio of a parameter is greater than the threshold, it indicates that this parameter has a significant impact on the performance index.

Taking the compressive strength as an example, the sensitivity order of the four process parameters on compressive strength i: clay content (13.158) > sintering temperature (2.631) > holding time (1.171) > molding pressure (0.812). It can be concluded that this order is consistent with that of the range analysis (Figure 9a). According to the significance results in Table 5 and Table 6, it can be seen that the clay content has a significant effect on the compressive strength of sintered ordinary bricks, sintering temperature and holding time have a significant effect on water absorption, and sintering temperature also has a significant effect on mass loss rate. The influence of all process parameters on bulk density of sintered ordinary bricks is not significant. Considering that the compressive strength of sintered ordinary brick is the main index affecting its performance, the clay content was selected to be 30%. The average compressive strength of sintered ordinary bricks with this clay content can meet the requirements of the highest strength grade, MU30, in the Chinese sintered ordinary bricks standard (GB/T 5101-2017), and also ensure large utilization of lead–zinc tailings. The optimal sintering temperature, holding time, and forming pressure are determined according to the parameter levels corresponding to the maximum value of each average strength curve. In summary, the optimal process parameters should be clay content of 30%, forming pressure of 20 MPa, sintering temperature of 1080 °C, and holding time of 60 min.

### 3.4. Verification and Mechanism Analysis

#### 3.4.1. Verification of Optimal Process Parameters

According to the optimal process parameters of sintered ordinary bricks with lead–zinc tailings determined in Section 3.3.3, corresponding specimens were prepared and their main performance indices are shown in Table 7. Compared with the results of the orthogonal experiment, the compressive strength of sintered brick with optimal process parameters is the highest, which fully meets the requirements of the highest strength grade, MU30, in the Chinese sintered ordinary bricks standard (GB/T5101-2017). According to Wei et al. [14], the optimal strength of sintered brick containing gold tailings is 22.45 MPa. It is clear that the strength of sintered brick fabricated in this study is higher. In terms of raw material proportions, 35% clay is used in the research of Wei et al. [14], and the clay content in this study is 30%. Fly ash and lead–zinc tailings can be regarded as solid waste. Therefore, the strength and utilization rate of the tailings sintered bricks fabricated in this study are competitive. For unfired bricks with lead–zinc tailings [25], the compressive strength reaches 10 MPa (MU10 grade) when the proportion of tailings is 45%. It can be seen that the mechanical properties of sintered bricks with lead–zinc tailings fabricated in this study are superior. In addition, the measured water absorption, mass loss rate, and bulk density of sintered ordinary bricks under this parameter combination also meet the requirements.

#### 3.4.2. Sintering Mechanism of Lead–Zinc Tailings

**Figure 10:**
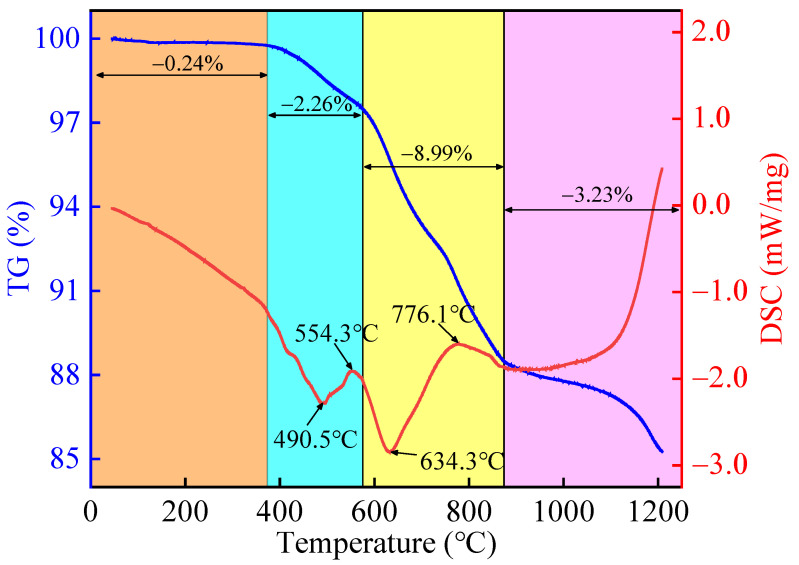
TG-DSC curve of lead–zinc tailings.

The thermogravimetry/differential scanning calorimetry (TG-DSC) curves of lead–zinc tailings are shown in Figure 10. It can be seen from the TG curve that lead–zinc tailings show a trend of weight loss during the sintering process, and the cumulative mass loss rate is 14.72%. The mass loss process can be divided into four stages: (1) The temperature rises from room temperature to around 370 °C during the first stage. The TG curve declines slowly in this period, and the cumulative mass loss rate is only 0.24%. The mass loss at this stage is mainly caused by the evaporation of adsorbed water and crystal water. However, the specimen was dried before thermal analysis, and part of the adsorbed and crystalline water was discharged, so the mass loss rate was small. (2) The second stage corresponds to the range in the sintering temperature from 370 °C to about 575 °C. The TG curve begins to decrease obviously, while the DSC curve shows obvious exothermic and endothermic peaks. The exothermic peak at about 490.5 °C may be caused by the combustion of organics contained in lead–zinc tailings, and the endothermic peak at about 554.3 °C is mainly related to the decomposition of pyrite and silicate, and is also affected by the quartz phase change. The quality of quartz does not change during the phase change, but the combustion of organic matter, and the decomposition of pyrite and silicate, will cause mass loss. (3) In the third stage, the sintering temperature rises from 575 °C to 870 °C. The DSC curve shows an obvious peak, and mass loss rate of lead–zinc tailings at this stage is the largest, about 8.99%. It is speculated that the exothermic peak is caused by the further oxidation of pyrite decomposition products, and the endothermic peak is mainly caused by the decomposition of carbonate minerals in lead–zinc tailings. Due to the high content of carbonate minerals in lead–zinc tailings, the mass loss increases significantly at this stage. (4) The sintering temperature above 870 °C corresponds to the fourth stage. In the early part of this stage, the mass loss is relatively slow, and the DSC curve also increases slowly, indicating that the internal changes in lead–zinc tailings are mainly melting, which stay in a stable sintering process. However, when the sintering temperature is higher than 1150 °C, the mass loss rate of tailings is intensified, and there exists an endothermic peak, which may be caused by the decomposition of aluminosilicate minerals.

#### 3.4.3. XRD and SEM Analysis

**Figure 11:**
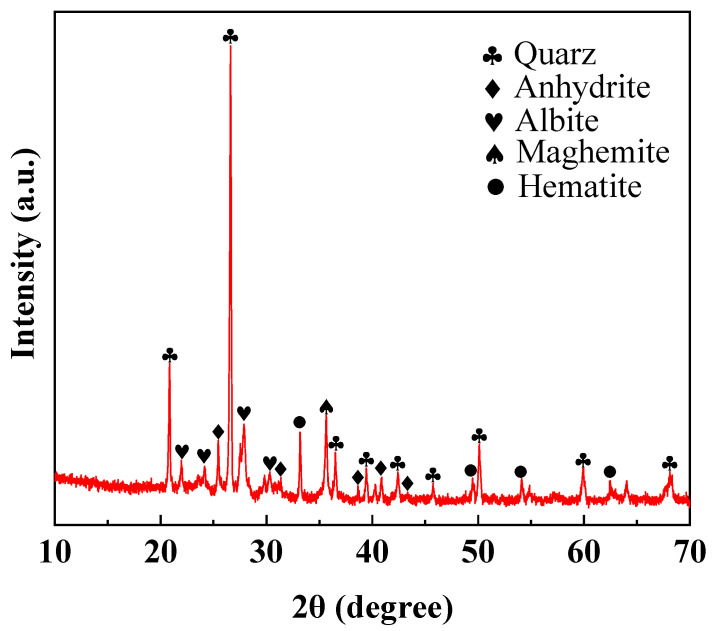
XRD pattern of sintered brick with optimal process parameters.

XRD and SEM experiments were carried out on sintered ordinary bricks prepared under the optimal combination of process parameters. The phase compositions of sintered ordinary brick are shown in Figure 11. It can be found that the main minerals in sintered ordinary bricks from lead–zinc tailings are quartz, anhydrite, albite, maghemite, and hematite. Comparing pre- and post-sintering XRD patterns (Figure 2 and Figure 11), it can be seen that the diffraction peaks of mica, dolomite, pyrite, chlorite, and calcite in lead–zinc tailings disappeared, while the diffraction peaks of anhydrite, albite, maghemite, and hematite appeared. This indicates that chemical reactions occurred in minerals during the sintering process. According to Table 2, raw materials contain *Na*_2_*O*, *Al*_2_*O*_3_, and *SiO*_2_, which can be used to form *NaAlSi*_3_*O*_8_. Mica (*KAl*_2_*(AlSi*_3_*O*_10_*)(OH)*_2_) is mainly decomposed into *SiO*_2_, *Al*_2_*O*_3_, *K*_2_*O*, and *H*_2_*O*. Dolomite is decomposed into *CaO*, *MgO*, and *CO*_2_. Pyrite is oxidized to *Fe*_2_*O*_3_ and *Fe*_3_*O*_4_, and then *Fe*_3_*O*_4_ is eventually oxidized to *γ-Fe*_2_*O*_3_ (maghemite) during the cooling process (around 220 °C). Chlorite (*Al*_4_*Si*_4_*O*_10_*(OH)*_8_) is decomposed into *Al*_2_*O*_3_, *SiO*_2_, and *H*_2_*O*, and calcite is decomposed into *CaO* and *CO*_2_. The decomposed *CaO* reacts with *SO*_2_ to form *CaSO*_3_, which is further oxidized to *CaSO*_4_. The minerals in the sintered bricks are responsible for the mechanical properties. The mica in lead–zinc tailings is decomposed into *SiO*_2_ and *Al*_2_*O*_3_ during the sintering process, which participate in the formation of quartz and albite to improve the uniaxial compressive strength of bricks. The relevant chemical equations are expressed as:

(1) $$CaMg{\left(CO_{3}\right)}_{2}(Dolomite)\to CaO+MgO+CO_{2}↑$$

(2) $$KAl_{2}\left(AlSi_{3}O_{10}\right){\left(OH\right)}_{2}(Mica)\to SiO_{2}+Al_{2}O_{3}+K_{2}O+H_{2}O↑$$

(3) $$FeS_{2}(Pyrite)+O_{2}\to Fe_{2}O_{3}(Hematite)/{\mathrm{Fe}}_{3}O_{4}+SO_{2}↑$$

(4) $$Fe_{3}O_{4}+O_{2}\to \gamma \text{-}Fe_{2}O_{3}(Maghemite)$$

(5) $$Al_{4}Si_{4}O_{10}{\left(OH\right)}_{8}(Chlorite)\to Al_{2}O_{3}+SiO_{2}+H_{2}O↑$$

(6) $$CaCO_{3}(Calcite)\to CaO+CO_{2}↑$$

(7) $$CaO+SO_{2}\to CaSO_{3}$$

(8) $$CaSO_{3}+O_{2}\to CaSO_{4}(Anhydrite)$$

**Figure 12:**
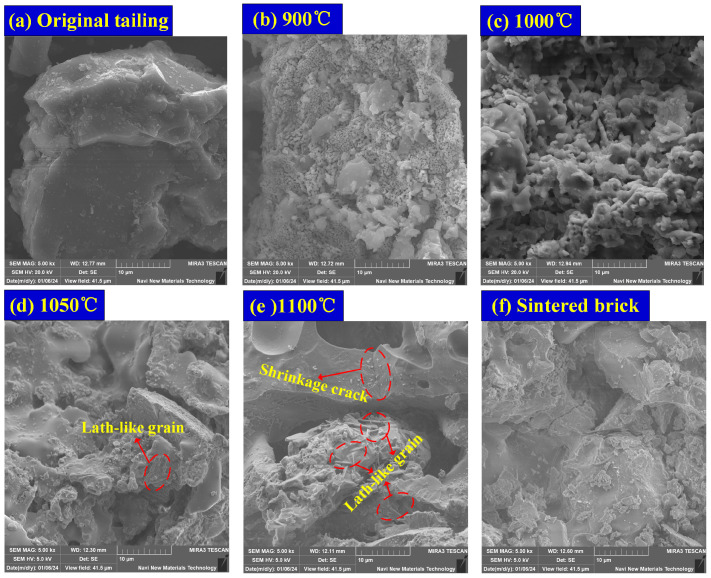
SEM images of lead–zinc tailings and sintered brick: (**a**) raw lead–zinc tailings; (**b**–**e**) lead–zinc tailings at sintering temperature of 900 °C, 1000 °C, 1050 °C, and 1100 °C, respectively; (**f**) sintered brick with optimal process parameters.

Figure 12 shows SEM images of raw tailings, sintered tailings at different temperatures, and sintered bricks fabricated according to the optimal process parameters (all magnified by 5000 times). By comparison, raw tailings are characterized by a single particle with flaky debris attached to its surface. From Figure 12a to Figure 12b, at a sintering temperature of 900 °C, the components of lead–zinc tailings are decomposed, the boundary of particles is no longer clear, the surface is rough and presents dense tentacle-like particles, and the micro-pores are very dense. Furthermore, there also exist large cloud-like particles. From Figure 12b to Figure 12c, it can be found that short tentacle-like particles almost disappear, which are replaced by cloud-like particles. Part of the lead–zinc tailings begins to turn into molten liquid, but the amount is small, and micro-pores are still dense. From Figure 12c to Figure 12d, sintered tailings at 1050 °C no longer show a uniform and dense porous structure, mainly because of the increasing molten liquid part, and the generated adhesive material further fills the pores. In addition, the cloud-like particles transform into smaller flocculent particles, and the crystalline boundary appears but is not smooth enough. Lath-like particles also appear, which is due to the high temperature calcination; the particles are fully developed through each other, the crystallization rate is accelerated, and eventually lead to the formation of prismatic crystal phase. From Figure 12d to Figure 12e, when the sintering temperature rises to 1100 °C, it can be seen that the number of lath-like crystals increases greatly, the surface is smoother, and there is less flocculent debris. In addition, micro-cracks can be observed in the figure, which are possibly shrinkage cracks created under high-temperature sintering. The SEM image of sintered brick fabricated according to the optimal process parameters is shown in Figure 12f. It can be seen that the surface of the aggregate is smooth and has a relatively obvious boundary, and the pores are very few, which is mainly attributed to the addition of clay and fly ash to fill the pores in the sintering process. In addition, micro-cracks also appear in the SEM image of the sintered brick, which may not be shrinkage cracks, but may also be caused by the force generated in the loading experiment or grinding. In general, in the range of 900 °C to 1100 °C, with the increase in sintering temperature, the reaction and crystallization of lead–zinc tailings particles will be more adequate, there will be fewer micro-pores, and the particle boundary will be smoother and clearer. Based on the SEM images, it can be determined that the optimal sintering temperature should be above 1050 °C, which also proves the correctness of setting the optimal sintering temperature to 1080 °C.

## 4. Conclusions

In this study, lead–zinc tailings were used as the main material, and clay and fly ash as the auxiliary materials, to prepare sintered bricks. Firstly, the effect of clay content on the properties of sintered brick was studied via the single-factor test. On this basis, taking clay content, molding pressure, sintering temperature, and holding time as the influencing factors, an orthogonal experiment was carried out to obtain the optimal process parameters. The main conclusions are as follows:

1. The single-factor experiment shows that with the increase in clay content, uniaxial compressive strength and bulk density increase continuously, while water absorption and mass loss rate decrease gradually. When the clay content is near 30%, the performance of sintered ordinary brick is better.
2. Based on the orthogonal experiment, the mean value analysis shows that the compressive strength increases with the increase in clay content, but increases first and then decreases with the increase in other parameters. Water absorption decreases with the increase in the four parameters, and is significantly affected by sintering temperature. The range analysis shows that the clay content and sintering temperature are the main factors affecting the performance of sintered brick. The variance analysis shows that clay content is a significant factor affecting the compressive strength, while sintering temperature and holding time are significant factors affecting water absorption.
3. According to the XRD pattern, during the sintering process, mica, pyrite, chlorite, calcite, and dolomite are decomposed to form hematite, maghemite, anhydrite, and albitite. Among them, mica with poor hardness is decomposed to SiO_2_ and Al_2_O_3_, which participate in the formation of quartz and albite with higher hardness. SEM images show that when the sintering temperature reaches more than 1050 °C, the crystallization degree of sintered lead–zinc tailings is higher, the surface is smoother and denser, and the porosity is reduced.
4. The optimal process parameters were obtained. That is, the raw material ratio is lead–zinc tailings:clay:fly ash = 6:3:1, molding pressure is 20 MPa, sintering temperature is 1080 °C, and holding time is 60 min. Under this condition, the compressive strength of sintered bricks is 34.94 MPa, which meets the requirements of the highest strength grade of MU30 in “Chinese Sintered Ordinary Bricks” (GB/T5101-2017). The sintered bricks with lead–zinc tailings can be used as pavement bricks around mining areas.

This study only investigated the compressive strength and other physical properties of sintered bricks with lead–zinc tailings at room temperature. In fact, the stress conditions and service environment of bricks are complex, and this manuscript does not consider the fatigue characteristics of bricks (used as road bricks) and durability. Mines are often located in high-altitude areas with harsh weather conditions, so the freeze–thaw resistance and optimization of sintered bricks with tailings can be developed in the future. Furthermore, considering environmental protection and energy conservation, the life-cycle management of bricks with lead–zinc tailings in an environmental context, and determining how to reduce energy consumption while fabricating bricks that fulfill the strength requirement, are of practical significance.

## Figures and Tables

**Table 1:** Mass ratio of different raw materials (unit: %).

Specimen ID	Lead–Zinc Tailings	Clay	Fly Ash
10-0-0	100	0	0
9-0-1	90	0	10
8-1-1	80	10	10
7-2-1	70	20	10
6-3-1	60	30	10
5-4-1	50	40	10

**Table 2:** Process parameters and corresponding values of orthogonal experiment.

Level	A: Clay Content (%)	B: Forming Pressure (MPa)	C: Sintering Temperature ( °C)	D: Holding Time (min)
1	20	15	1050	30
2	25	20	1080	60
3	30	25	1110	90
4	35	30	1140	120

**Table 3:** Main chemical compositions of raw materials (unit: %).

Materials	SiO_2_	Al_2_O_3_	Fe_2_O_3_	MgO	CaO	K_2_O	P_2_O_5_	Na_2_O	MnO_2_	TiO_2_	ZnO	Others
Tailings	48.17	10.79	14.15	4.14	4.20	3.01	/	0.456	0.73	0.312	0.493	13.55
Clay	61.37	14.32	4.74	2.36	12.40	2.59	0.21	1.03	/	/	/	0.98
Fly ash	48.80	26.26	4.87	1.84	4.95	2.00	0.15	1.67	/	/	/	9.46

**Table 4:** Performance indices of sintered ordinary bricks from lead–zinc tailings.

Specimen ID	Compressive Strength/MPa	Water Absorption/%	Mass Loss Rate/%	Bulk Density/(g/cm^3^)
A_1_B_1_C_1_D_1_	9.40	18.38	7.64	1.69
A_1_B_2_C_2_D_2_	25.37	17.16	9.60	1.73
A_1_B_3_C_3_D_3_	22.91	14.92	10.75	1.74
A_1_B_4_C_4_D_4_	11.16	4.26	10.40	1.68
A_2_B_1_C_2_D_3_	23.50	18.48	10.09	1.67
A_2_B_2_C_1_D_4_	25.65	16.97	10.03	1.71
A_2_B_3_C_4_D_1_	16.33	9.55	10.84	1.76
A_2_B_4_C_3_D_2_	22.6	14.57	10.64	1.73
A_3_B_1_C_3_D_4_	32.17	12.39	10.97	1.79
A_3_B_2_C_4_D_3_	26.15	4.62	11.15	1.76
A_3_B_3_C_1_D_2_	32.01	16.67	9.20	1.73
A_3_B_4_C_2_D_1_	30.66	15.99	9.20	1.76
A_4_B_1_C_4_D_2_	31.89	6.08	10.84	1.81
A_4_B_2_C_3_D_1_	34.43	16.55	10.10	1.71
A_4_B_2_C_2_D_4_	33.96	15.20	10.18	1.71
A_4_B_4_C_1_D_3_	31.95	16.53	9.33	1.74

**Table 5:** Variance of parameters’ influences on compressive strength and water absorption.

Performance Indices	Parameters	Sum of Square	Degree of Freedom	F-Ratio	Threshold of F-Ratio (α = 0.05)	Significance
Compressive strength	Clay content	641.670	3	13.158	9.280	Significant
	Forming pressure	39.604	3	0.812		None
	Sintering temperature	128.322	3	2.631		None
	Holding time	57.112	3	1.171		None
	Error	48.77	3			
Water absorption	Clay content	12.269	3	7.360	9.280	None
	Forming pressure	3.653	3	2.191		None
	Sintering temperature	316.063	3	189.600		Significant
	Holding time	16.970	3	10.180		Significant
	Error	1.67	3			

**Table 6:** Variance of parameters’ influences on mass loss rate and bulk density.

Performance Indices	Parameters	Sum of Square	Degree of Freedom	F-Ratio	Threshold of F-Ratio (α = 0.05)	Significance
Mass loss rate	Clay content	1.349	3	4.557	9.280	None
	Forming pressure	0.470	3	1.588		None
	Sintering temperature	7.890	3	26.655		Significant
	Holding time	2.254	3	7.615		None
	Error	0.30	3			
Bulk density	Clay content	0.006	3	0.667	9.280	None
	Forming pressure	0.000	3	0.000		None
	Sintering temperature	0.004	3	0.444		None
	Holding time	0.002	3	0.222		None
	Error	0.01	3			

**Table 7:** Performance indices of sintered brick with optimal process parameters.

Performance Indices	Value
Compressive strength	34.94 MPa
Water absorption	16.02%
Mass loss rate	9.85%
Bulk density	1.75 g/cm^3^

## Data Availability

The raw data supporting the conclusions of this article will be made available by the authors on request.
